# Influence of long-acting growth hormone analogs on other hypothalamic-pituitary axes: a real-world study

**DOI:** 10.1007/s40618-026-02986-1

**Published:** 2026-07-29

**Authors:** Giulia Rodari, Eriselda Profka, Aurora Pedroli, Valeria Citterio, Valentina Collini, Camilla Mazzi, Giovanna Mantovani, Claudia Giavoli

**Affiliations:** 1 Endocrinology Unit, Fondazione IRCCS Ca’ Granda Ospedale Maggiore Policlinico, Milan, Italy; 2 Department of Clinical Sciences and Community Health, Department of Excellence 2023- 2027, University of Milan, Milan, Italy

**Keywords:** Central hypothyroidism, Central hypoadrenalism, GH deficiency, Long-acting growth hormone

## Abstract

**Introduction/purpose:**

Long‑acting growth hormone (LAGH) formulations have emerged to address poor adherence to daily recombinant human growth hormone (rhGH), offering reduced injection frequency and comparable efficacy. Though their efficacy and safety in children have been demonstrated in registrative trials and in some real-world studies, their impact on other pituitary axes remains unclear.

**Methods:**

This observational prospective monocentric study explores hormonal changes in isolate growth hormone deficiency (GHD) children (*n* = 33) starting LAGH Somatrogon (*n* = 16, G1) or switching from daily rhGH to the same weekly analogue (*n* = 17, G2). In all study patients, baseline and post‑treatment assessments (after 3–6 months of weekly LAGH start) included IGF‑I, ACTH, cortisol, TSH, FT4 and FT3 levels.

**Results:**

After LAGH initiation, IGF‑I SDS increased significantly in both groups (*p* < 0.001), more frequently exceeding 2 SDS in G2 (6/17 vs. 2/16 in G1). No significant changes occurred in TSH, FT3, FT4, ACTH or cortisol levels after LAGH start, nor between‑group differences.

**Conclusions:**

In isolated GHD, adrenal and thyroid function remained stable after LAGH initiation, with comparable results in treatment‑naïve patients and in those already receiving daily therapy. A greater increase in IGF-I levels was observed in switchers, suggesting that a lower starting dose may be appropriate in patients previously treated with rhGH. Further studies are required to clarify the endocrine effects of long‑acting formulations in individuals with associated multiple pituitary hormone deficiencies.

## Introduction

Daily recombinant human growth hormone (rhGH) is currently approved for use in children and adults with growth hormone deficiency (GHD) in many countries and has a relatively favourable tolerability profile. The efficacy and safety of daily rhGH administration have been extensively documented [1–6]. However, as the number of clinical outcome reports in patients treated with daily rhGH has increased over the years, low adherence to therapy has emerged as a significant factor contributing to suboptimal treatment response [7–10]. Daily injections require considerable effort from patients and caregivers, which can lead to a decline in adherence over time. Long-acting growth hormone (LAGH) formulations have been proposed as a potential alternative to reduce the treatment burden on patients and their families by decreasing the frequency of injections, ultimately aiming to improve treatment outcomes [11].

Long‑acting GH (LAGH) formulations achieve extended duration through distinct molecular strategies. For instance, Lonapegsomatropin is a prodrug in which GH is transiently PEGylated. Somapacitan contains a single‑point mutation in the GH backbone (amino acid 101) and incorporates a non‑covalent albumin‑binding moiety. Somatrogon is a GH fusion protein containing three carboxy‑terminal peptides derived from human chorionic gonadotropin. Somapacitan and Somatrogon are administered via a pen device that allows adjustable dosing and multiple doses per pen, whereas Lonapegsomatropin is delivered through an autoinjector with fixed‑dose cartridges. All three formulations are administered once weekly with weight‑based dosing. Differences in dose per kilogram reflect the distinct molecular weights and pharmacokinetic–pharmacodynamic properties of each molecule [11].

These LAGH analogues are attracting as they may theoretically improve patient acceptance, tolerability, and offer greater therapeutic flexibility. However, potential drawbacks exist, such as an unphysiological GH profile and differences in molecular structure, which could present clinical challenges related to dose initiation and titration, therapeutic monitoring, side effect incidence and duration, as well as long-term safety [11].

Additionally, fluctuations in peak and trough serum GH and IGF-I levels, along with variations in therapeutic efficacy and safety, may be influenced by the specific technology used to extend GH action. In this context, weekly Somatrogon shows the expected pharmacodynamic pattern, with IGF‑I levels rising to a post‑dose peak (2–2.5 days post-injection) and subsequently declining over the course of the weekly interval. As mean weekly IGF‑I concentration represents the most clinically relevant measure of systemic exposure to once‑weekly Somatrogon [12], and given that values obtained 96 h after dosing most accurately approximate this parameter [13], IGF‑I determinations should be systematically conducted on Day 4 [12].

Pharmacological studies on some LAGH analogues, i.e. Somatrogon, have demonstrated non-inferiority compared to daily rhGH, showing comparable increases in growth velocity in children [14–17] and improvements in body composition in adults [18, 19] with GHD, without any significant or unexpected adverse events [15–17].

Nevertheless, several practical questions remain unanswered, including potential influences on the broader endocrine networks.

Indeed, untreated GHD may conceal concomitant pituitary hormone deficiencies-most commonly central hypothyroidism and central adrenal insufficiency-because the clinical and biochemical manifestations of these conditions can be attenuated in the setting of GH/IGF‑I axis impairment [20]. In such cases, initiation of rhGH therapy may unmask previously unrecognized deficits. Consistently, the Summary of Product Characteristics for some long‑acting GH analogues (e.g., Somatrogon ) recommends reassessing thyroid and adrenal function after treatment initiation in all patients, including those with isolated GHD, although these recommendations are largely based on evidence from studies conducted with daily rhGH formulations. Indeed, no real-world data are currently available on the effects of LAGH on other pituitary axes.

The aim of this study is to evaluate the impact of LAGH on hypothalamic-pituitary-thyroid and -adrenal axis as well as the increase in IGF-I concentrations, in children with GHD following the initiation of weekly GH analogues or after switching from daily GH to LAGH preparations.

## Materials and methods

### Patients and study protocol

The study included 33 children (M/F 22/11) with isolated GHD followed at the Endocrinology Unit of Fondazione IRCCS Ca’ Granda Ospedale Maggiore Policlinico of Milan. At the time of diagnosis, all patients fulfilled the auxological criteria of height (HT) ≤ -3 SDS or HT ≤ -2 SDS with a growth velocity (GV) ≤ -1 SDS or a GV ≤ -2 SDS for the last year or GV ≤ -1.5 SDS for two consecutive years. All patients underwent to arginine and glucagon test. In prepubertal boys older than 11 years and prepubertal girls older than 10 years, estradiol valerate (2 mg) was administered for two days before the provocative test [21]. According to the biochemical diagnostic criteria defined by AIFA Note 39-the Italian Medicines Agency’s regulatory framework governing the prescription and reimbursement of GH therapy-GHD was diagnosed when both provocative tests showed a GH peak below 8 µg/L [22]. Upon diagnosis of GHD, each patient underwent an MRI of the brain and of the hypothalamic-pituitary region. At baseline age, mid-parental height (MPH), height (HT), weight, body mass index (BMI) and pubertal stage were recorded (Tanner stages and testicular volume throughout Prader’s orchidometrer) and SDS values for HT and MPH and body mass index were calculated according to WHO growth charts [23].

All patients born small for gestational age, with underlying genetic/chronic diseases (i.e. Prader–Willi syndrome, SHOX deficiency, chronic kidney disease or coeliac disease) or concomitantly treated with drugs negatively influencing growth, were excluded.

Study patients included two groups: Group 1 (G1, *n* = 16) treatment-naïve GHD patients initiating therapy with the LAGH formulation Somatrogon and Group 2 (G2, *n* = 17) GHD children switched from daily to the same weekly formulation (Somatrogon), upon patient or family request. Both at baseline (T0) and after at least 3–6 months of LAGH therapy (T1) at the initial dose of 0.66 mg/kg/week, the following values were collected: insulin-like growth factor I (IGF-I), ACTH, cortisol, thyrotropin hormone (TSH), free-levothyroxine (FT4), free- triiodothyronine (FT3). For all study patients, IGF‑I values were consistently measured on the fourth day after Somatrogon administration, as this time point best approximates mean weekly IGF‑I concentrations and therefore reflects systemic exposure to once‑weekly Somatrogon [24]. Values were compared with age‑ and sex‑adjusted reference ranges for each assay and expressed as standard deviation scores (SDS), to support interpretation.

All procedures performed in this study were in accordance with the ethical standards of the institutional research committee and with the 1964 Helsinki declaration and its later amendments or comparable ethical standards. Informed written consent was obtained from all parents and/or a legal guardian.

### Statistical analysis

In order to define the population characteristics, descriptive statistics has been used (mean ± SD for normally distributed variables and median-interquartile range - IQR for the others). To compare normally distributed variables with homogeneous variances, the Student’s t‑test was used, whereas the Mann–Whitney test was applied for non‑normally distributed variables. In addition, when analysing paired data, both parametric (paired Student’s t‑test) and non‑parametric (Wilcoxon signed‑rank test) methods were performed as appropriate. Categorical variables were compared using the χ2 or Fisher test.

Statistical significance has been set for *p* values < 0.05.

Statistical analysis has been performed using STATA software version 17 (College Station, Texas 77845 USA).

## Results

### Baseline characteristics

Median age at GHD diagnosis was 10.5 years (IQR 7.4 ‒ 11.3), being not significantly different in the two groups (*p =* 0.769).

Among the whole cohort of 33 children, 22 (66.7%) patients were males (M/F 12/4 in G1 and 10/7 in G2). The baseline characteristics at GHD diagnosis were not different in the two groups as depicted in Table 1. At diagnosis, the majority of patients was pre-pubertal (22/33, 66.7%, Table 2).

**Table 1:** Auxological and biochemical data at GHD diagnosis in the whole cohort (*n* = 33) and stratified by subgroup

Variable	*N* = 33 Mean (SD); Median (IQR)	Group 1 (*N* = 16) Mean (SD); Median (IQR)	Group 2 (*N* = 17) Mean (SD); Median (IQR)	*p*-value
CA^1^ (years)	10.5 (7.4 ‒ 11.3)	10.3 (6.8 ‒ 11.6)	10.5 (8.3 ‒ 11.3)	ns*
MPH^2^ (cm)	170.8 (7.8)	172.8 (9.7)	169.2 (5.5)	ns°
MPH^2^ (SDS^3^)	0.1 (0.8)	0.1 (1.0)	0.1 (0.5)	ns°
HT^4^ (cm)	127.0 (112.5 ‒ 132.1)	124 (103 ‒ 131)	127 (125 ‒ 141)	ns*
HT^4^ (SDS)	-2.2 (-2.4 ‒ -1.5)	-2.3 (-2.5 ‒ -1.5)	-2.1 (-2.4 ‒ -0.8)	ns *
BMI^5^ (kg/m2)	15.3 (14.2 ‒ 17.4)	15.2 (14.2 ‒ 15.4)	17.3 (14.5 ‒ 17.9)	ns*
BMI^5^ (SDS^3^)	0.1 (-1.4 ‒ 0.6)	-0.72 (-1.39 ‒ 0.5)	0.29 (-1.59 ‒ 0.82)	ns*
GV^6^ (cm/years)	3.7 (1.2)	3.8 (1.6)	3.6 (0.8)	ns°
GV^6^ (SDS^3^)	-2.1 (-2.8 ‒ -1.8)	-2.0 (-2.7 ‒ -1.8)	-2.1 (-3.0 ‒ -1.7)	ns*
Max GH^7^ peak (mcg/dL)	4.9 (1.9)	5.3 (1.7)	4.6 (2.1)	ns°
IGF-I^8^ (ng/mL)	101.0 (32.7)	101.3 (32.3)	100.7 (34.1)	ns°
IGF-I^8^ (SDS^3^)	-1.6 (0.6)	-1.5 (0.7)	-1.6 (0.5)	ns°
TSH^9^ (mIU/mL)	2.1 (1.2 ‒ 3.1)	1.8 (1.2 ‒ 2.4)	2.4 (1.4 ‒ 3.7)	ns*
FT4^10^ (ng/L)	12.1 (1.2)	12.7 (1.0)	11.5 (1.2)	ns°
FT3^11^ (ng/L)	4.1 (0.4)	4.1 (0.4)	3.8 (0.2)	ns°
ACTH^12^ (pg/mL)	20.3 (15.1 ‒ 34.0)	21.05 (14.4 ‒ 33.4)	20.3 (17.2 ‒ 34)	ns*
Cortisol (mcg/dL)	12.7 (8.9 ‒ 14.4)	12.5 (7.6 ‒ 14.5)	13 (9.1 ‒ 14.4)	ns*

CA^1^: Chronological Age; MPH^2^: Mid-parental Height; SDS^3^: Standard deviation score; HT^4^: Height; BMI^5^: Body Mass Index; GV^6^: Growth velocity; GH^7^: Growth Hormone; IGF-I^8^: Insulin-like Growth Factor I; TSH^9^: thyrotropin hormone, FT4^10^: Free-thyroxine; FT3^11^: Free-triiodothyronine (FT3), ACTH^12^: adrenocorticotropin hormone

°Unpaired t-Student test

*Mann–Whitney test

**Table 2:** Tanner stages at diagnosis

Tanner Stage	Group 1 (*n*, %)	B^1^/G^2^ (*n*)	TV^3^ (mL)	Group 2 (*n*, %)	B^1^/G^2^ (*n*)	TV^3^ (mL)
1	10 (62.5)	3/7	2	12 (70.6)	6/6	2
2	5 (31.25)	1/4	4	4 (23.5)	1/3	4
3	1 (6.25)	0/1	5	1 (5.9)	0/1	5

B^1^: Breast stage; G^2^: Genitals; TV^3^: Testicular volume

Brain MRI was normal in most of the patients (23/33, 70%, 12 in G1 and 11 in G2, respectively), whereas 24% of patients (8/33, 4 in G1 and 4 in G2, respectively) had an anterior pituitary hypoplasia and 2/33 (6%, all G2 patients) had pituitary stalk interruption syndrome.

Long-acting growth hormone was started at 0.66 mg/kg/day in all study patients. Among children in G2, the median daily rGH dose prior to switching to LAGH was 0.025 mg/kg/day (IQR 0.023–0.026 mg/kg/die).

Baseline auxological and biochemical data at GHD diagnosis are reported in Table 1.

At the time of GHD diagnosis, both in treatment‑naïve patients and in those already receiving rhGH, basal and glucagon‑stimulated cortisol levels were within the normal range, with peak values exceeding 14.6 mcg/dL [25]. Consistently, basal TSH and free thyroid hormone levels excluded a condition of central hypothyroidism.

**Fig. 1:**
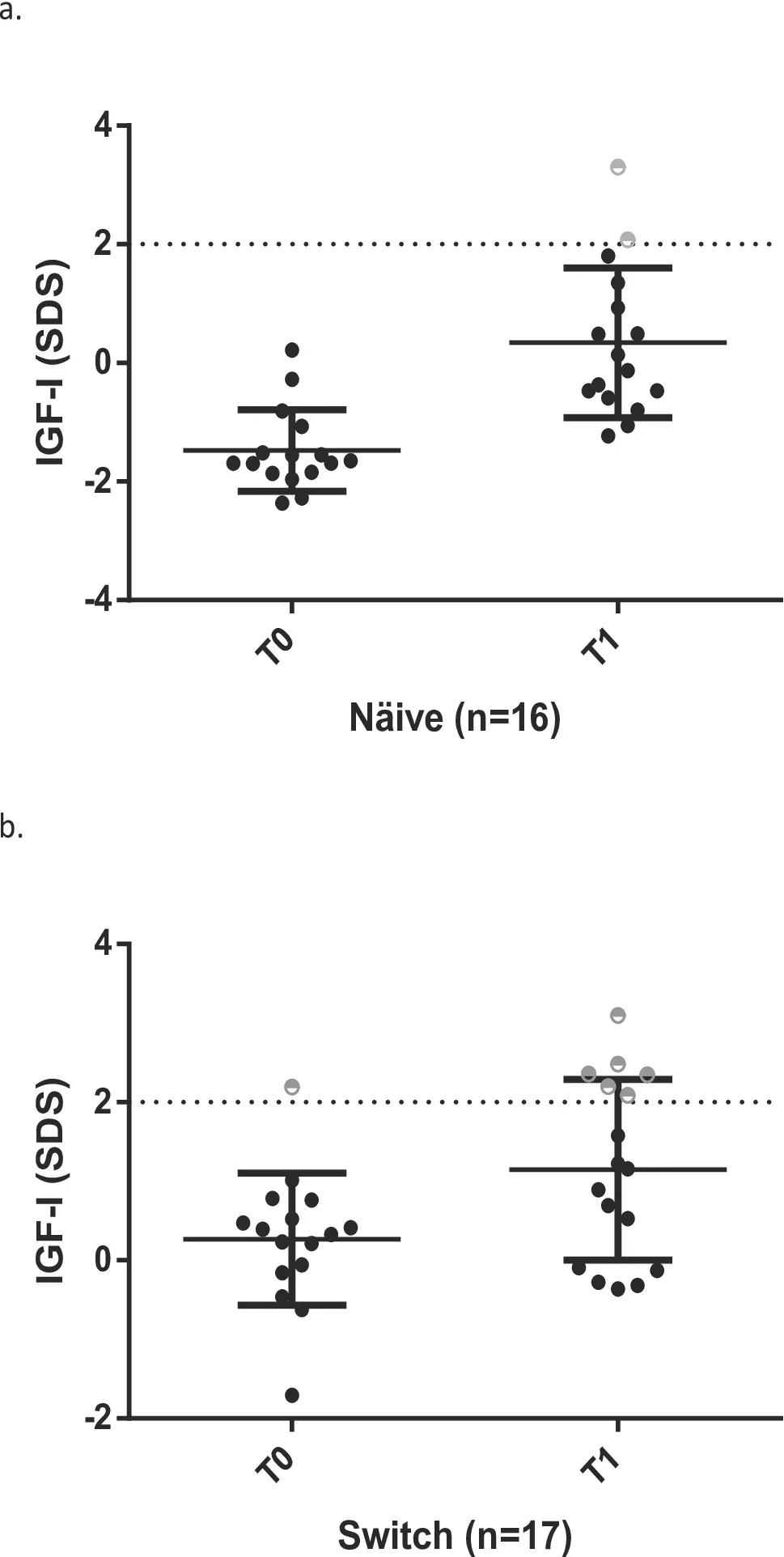
IGF-I levels before (T0) and after (T1) LAGH initiation in treatment-naïve (**a**) and patients switched from daily therapy (**b**)

After LAGH start, as expected, IGF-I SDS levels measured at day 4 after Somatrogon administration, significantly increased in G1 (*p* < 0.001), reaching values over 2 SDS in 2/16 cases (12.5%, Fig. 1a). Interestingly, even in patients of G2, switching from daily to weekly analogues, IGF-I SDS levels significantly increased (*p* = 0.015) reaching values over 2 SDS in 6/17 cases (35.3%, Fig. 1b), regardless pubertal stage.

On the other hand, no difference in ACTH or basal cortisol levels were observed after LAGH initiation in G1 (Table 3a) and G2 (Table 3b).

**Table 3:** Hormonal values before (T0) and after LAGH (T1) initiation in naïve G1 (a) and treated with rhGH G2 (b) children

(a)			
Naïve (G1, *n* = 16)			
Variable	T0	T1	*p*-sign
IGF-I^1^ (ng/mL)	101.3 (32.3)	263.4 (127.1)	< 0.001°
IGF-I^1^ (SDS^2^)	-1.5 (0.7)	0.3 (1.3)	< 0.001°
TSH^3−^ (mIU/mL)	1.8 (1.2 ‒ 2.4)	1.7 (1.5–2.3)	ns*
FT4^4^ (ng/L)	12.7 (1.0)	12.2 (2.0)	ns°
FT3^5^ (ng/L)	4.1 (0.4)	4.8 (0.7)	ns°
ACTH^6^ (pg/mL)	21.05 (14.4 ‒ 33.4)	23.4 (16.8 ‒ 27.5)	ns*
Cortisol (mcg/dL)	12.5 (7.0 ‒ 14.5)	11.5 (7.9 ‒ 14.1)	ns*

(b)			
Switch (G2, *n* = 17)			
Variable	T0	T1	*p*-sign
IGF-I^1^ (ng/mL)	318.2 (124.3)	393.4 (142.4)	0.011°
IGF-I^1^ (SDS^2^)	0.3 (0.8)	1.2 (1.2)	0.015°
TSH^3−^ (mIU/mL)	2.5 (1.6 ‒ 3.3)	2.0 (1.5 ‒ 2.7)	ns*
FT4^4^ (ng/L)	12.6 (2.2)	12.9 (1.6)	ns°
FT3^5^ (ng/L)	3.7 (0.8)	3.5 (0.2)	ns°
ACTH^6^ (pg/mL)	23.8 (18.7 ‒ 27.5)	22.8 (18.6 ‒ 29.4)	ns*
Cortisol (mcg/dL)	12.4 (7.7 ‒ 15.3)	10.3 (7.4 ‒ 13.4)	ns*

IGF-I^1^: Insulin-like Growth Factor I; SDS^2^: Standard deviation score; TSH^3^: thyrotropin hormone, FT4^4^: Free-thyroxine; FT3^5^: Free-triiodothyronine (FT3), ACTH^6^: adrenocorticotropin hormone

°Paired t-Student test

*Wilcoxon signed‑rank test

**Fig. 2:**
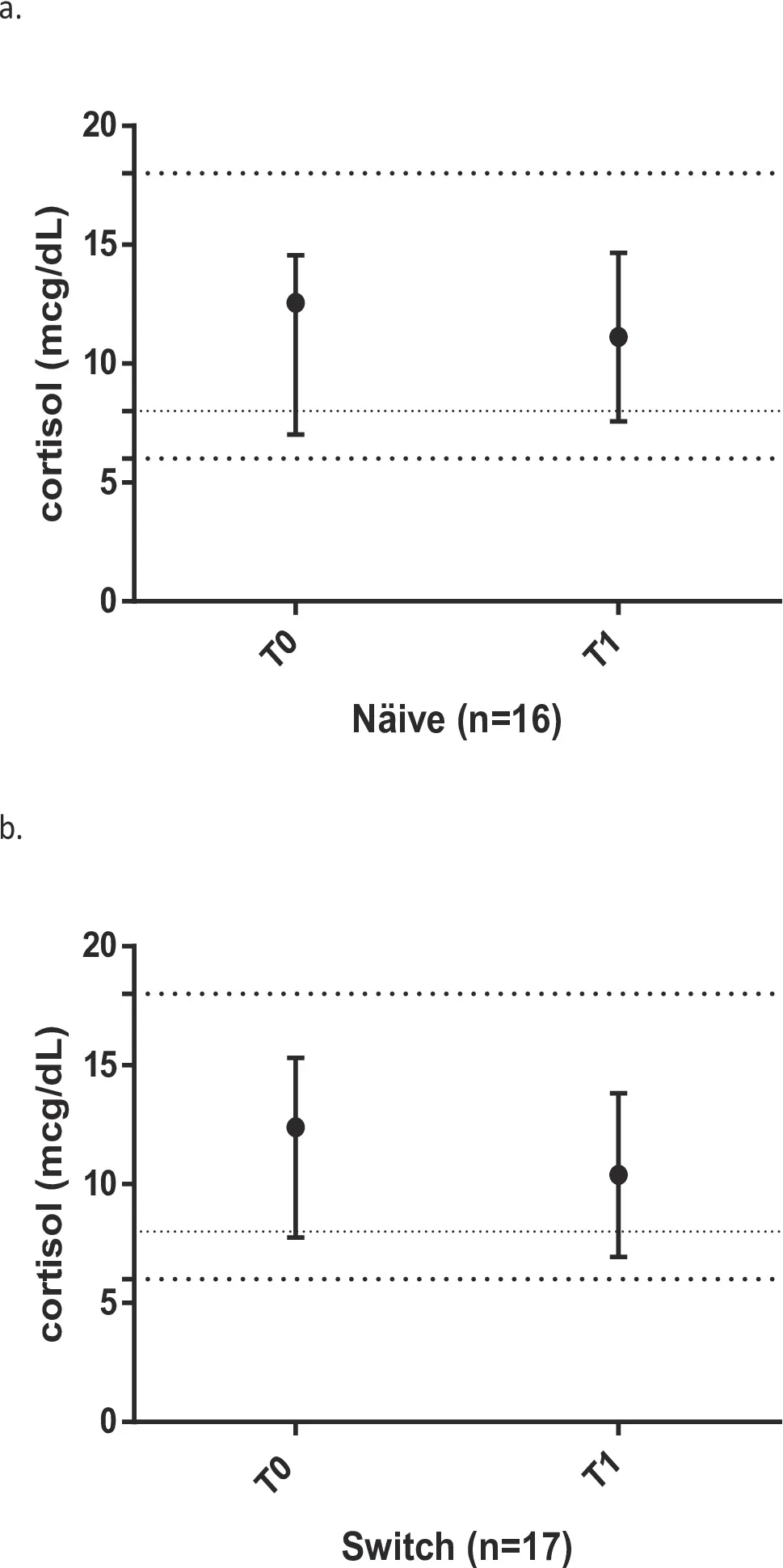
Cortisol levels before (T0) and after (T1) LAGH initiation in treatment-naïve (**a**) and patients switched from daily therapy (**b**)

Although not statistically significant, a reduction in basal cortisol levels was noted in 8/16 patients of G1 (Fig. 2a). In G2, despite ongoing GH replacement, basal cortisol levels decreased in 5/17 cases (Fig. 2b).

In patients with basal cortisol levels < 8 mcg/L (4/16 in G1, 3/17 in G2), a low-dose ACTH (1 mcg) stimulation test was performed, showing an adequate cortisol response (cortisol peak above 13.6 mcg/dL [26]).

**Fig. 3:**
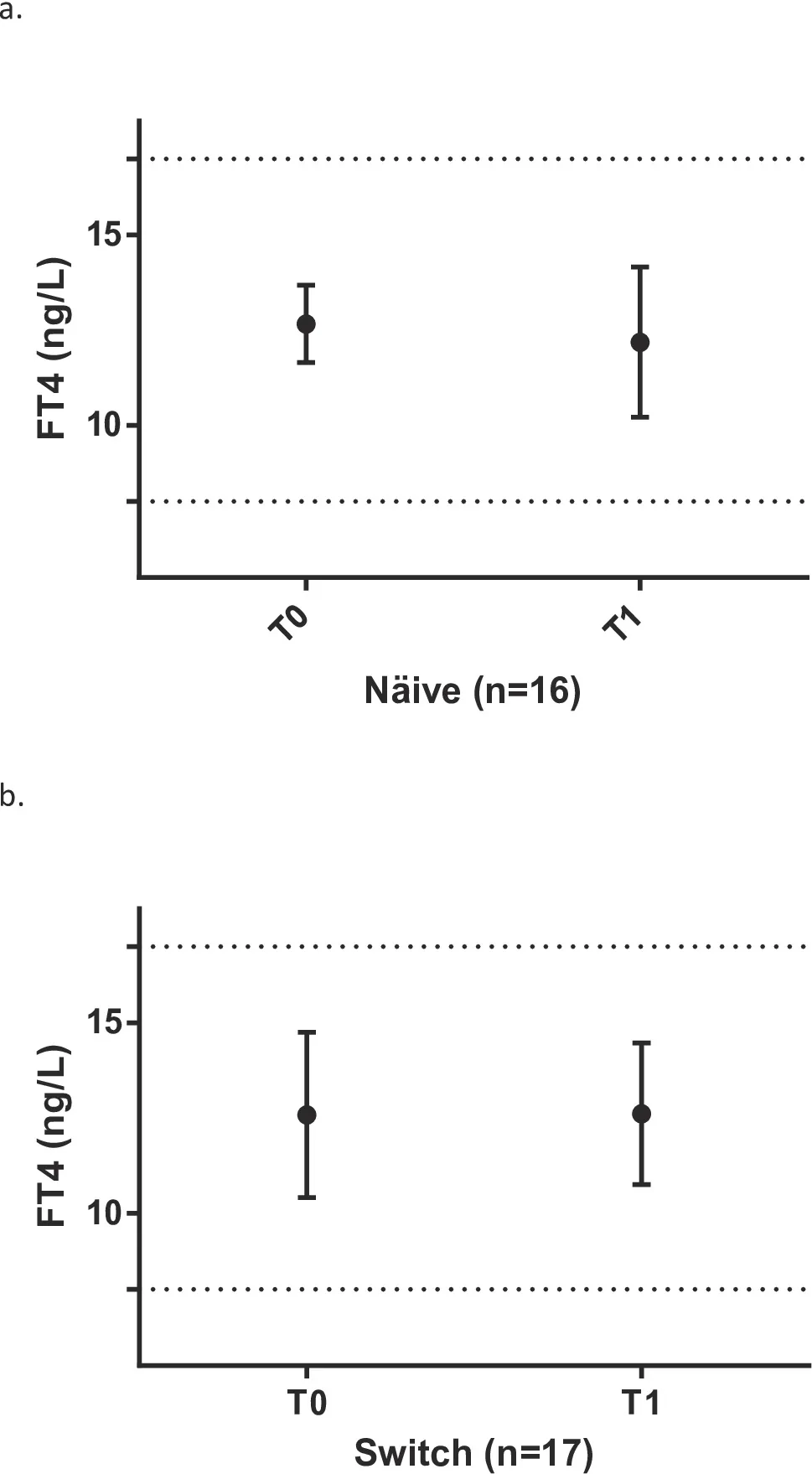
FT4 levels before (T0) and after (T1) LAGH initiation in treatment-naïve (a) and patients switched from daily therapy (b)

No difference was found in TSH, FT3 or FT4 levels before and after LAGH start either in G1 or G2 (Table 3). Although not statistically significant, FT4 levels decreased in 10/16 patients in G1, while remaining within the reference range (Fig. 3a). In G2, only 5/17 patients showed a reduction in FT4 levels after starting LAGH, despite already on GH replacement (Fig. 3b). Notably, patients with PSIS did not exhibit any decline in FT4 levels following LAGH initiation.

When comparing hormonal values after LAGH start between the two Groups, no significant difference was found in any considered variable. Nonetheless, it is worth noticing that IGF-I SDS values were higher in G2 than in G1, though not reaching statistically significance.

## Discussion

To the best of our knowledge, this is among the first studies to examine real‑world data on the endocrine effects of LAGH formulation Somatrogon in children with isolated GHD, with a specific focus on the hypothalamic-pituitary-adrenal (HPA) and –thyroid (HPT) axes.

Existing literature supports the presence of a complex interplay between the GH/IGF-I system and other hypothalamic–pituitary axes. Untreated GHD may mask an underlying ACTH deficiency. This phenomenon has significant clinical implications in patients with MPHD, who may experience a life-threatening adrenal crisis after rhGH initiation in the presence of an untreated central hypoadrenalism. These changes can be due, firstly, to a possible direct effect of GH on cortisol-binding globulin (CBG) at a peripheral level [20] and, secondly, to the modulating activity of 11beta-hydroxysteroid dehydrogenase.

(11ßHSD) at a tissue level, the well-known cortisol-cortisone shuttle [20]. Growth hormone, indeed, modulates cortisol metabolism mainly by inhibiting 11ßHSD1, thus leading to a reduced cortisone activation into cortisol. In GHD, this modulation is disrupted, leading to reduced conversion of cortisone to cortisol. Thus, higher cortisol levels may be present despite ACTH deficiency. After initiation of rhGH replacement, modulation of the GH/IGF-I axis on the cortisol–cortisone shuttle is restored, potentially resulting in decreased cortisol levels and unmasking a pre-existing central hypoadrenalism. Accordingly, in patients with suspected MPHD, current Guidelines recommend assessing the integrity of the HPA axis both prior (to avoid diagnostic pitfalls) and after rhGH start [21, 27]. On the contrary, in patients with isolated idiopathic GHD, no clinically significant impact has been reported in HPA axis [28]. Consistently, in our cohort of children with idiopathic isolated GHD, we could not find any significant difference between basal cortisol levels before and after LAGH start.

As far as hypothalamic-pituitary-thyroid axis is concerned, GH can influence the HPT axis and thyroid hormone concentrations through several different mechanisms. Peripherally, GH stimulates the extra-thyroidal conversion of thyroxine to the active hormone triiodothyronine (T3), thus reducing the inactive form reverse-T3(rT3) and increasing the T3/T4 ratio [20]. In MPHD, the type 2 deiodinase activity is usually upregulated to compensate the initial reduction in T4 by enhancing T3 production, whereas patients with hypothyroidism could lack of this compensatory mechanism [29].

Centrally, it has been postulated that GH can suppress TSH production due to an increased somatostatinergic tone or an increased T3 negative feedback within the thyrotropes, driven by increased T3 conversion from T4 deiodination [20]. In clinical practice, these mechanisms imply that rhGH therapy may unmask an underlying central hypothyroidism, making it essential to assess and monitor thyroid function both before and after starting rhGH [21], so that levothyroxine replacement can be initiated or adjusted if needed [27]. Notably, a decline in FT4 levels typically occurs within the first six months of rhGH therapy, highlighting the importance of early reassessment of thyroid function after treatment initiation [20], but can persist even afterwards [30]. In the present cohort of GHD children, no significant changed in TSH and FT4 occurred after LAGH start, neither in naïve patients, nor in those switching from daily to weekly preparations. These findings support previous evidence indicating that, as reported for rhGH, LAGH does not induce central hypothyroidism in the presence of an intact HPT axis.

Our study has several limitations that should be considered. Firstly, the small sample size, including only isolated GHD, mainly with normal brain MRI. In particular, the number of patients with PSIS was very limited, which restricts the robustness of subgroup analyses and prevents firm conclusions for this specific population.

Secondly, dynamic assessment of the HPA axis was not systematically performed. This was an intentional methodological choice, as stimulation testing was deemed unwarranted in the presence of acceptable basal cortisol concentrations and unremarkable clinical and biochemical parameters (including electrolytes and glucose). Although this approach is consistent with real‑world clinical practice, it precludes definitive conclusions regarding subtle impairments in adrenal reserve. Moreover, any potential unmasking of central adrenal insufficiency after GH initiation may involve only mild or borderline abnormalities, which would require dynamic testing or LC/MS‑based cortisol assessment to be reliably detected. Consequently, the absence of an observed effect of weekly GH treatment on adrenal function should be interpreted as a cautious inference based on the available data rather than a definitive conclusion.

Nonetheless, the presented data might be of clinical significance considering the exploratory design, firstly describing Somatrogon influences on HPA and HPT axes in children with isolated GHD.

In our cohort, IGF‑I responses showed a subtly divergent pattern between treatment‑naïve children and those switching from daily GH, despite initiation on the same Somatrogon dose. Switching patients exhibited a comparatively greater early rise in IGF‑I, whereas naïve patients demonstrated a more gradual increase. This observation suggests that children transitioning from daily to weekly GH may have a higher pharmacodynamic sensitivity to the initial Somatrogon dose, supporting the rationale for considering a lower starting dose in this subgroup to prevent the supraphysiological rise in IGF‑I, which on the contrary was rarely observed in naïve patients.

In conclusion, our findings provide an initial but essential step toward understanding how the use of LAGH formulation Somatrogon in GHD interact with the other hypothalamic-pituitary axes. To date, the Summary of Product Characteristics of LAGH analogues currently approved recommends reassessing these hormonal axes after treatment initiation, though considering results of studies conducted with daily rhGH formulations. According to present results, and as expected in isolated GHD, no significant differences were found between pre‑ and post‑LAGH HPA and HPT values, neither in naïve patients nor in those switching from daily rhGH to Somatrogon.

Further studies are needed to better explore the impact of LAGH therapy on other hypothalamic–pituitary axes in patients with MPHD. Moreover, additional insight into IGF‑I dynamics and glucose metabolism after LAGH initiation is required, particularly to clarify differences between LAGH‑naïve patients and those switching from daily rhGH.
